# Transcatheter Arterial Chemoembolization of Hepatocellular Carcinoma as a Bridge to Liver Transplantation: A Retrospective Study

**DOI:** 10.4061/2011/974514

**Published:** 2011-01-17

**Authors:** Antoine Bouchard-Fortier, Réal Lapointe, Pierre Perreault, Louis Bouchard, Gilles Pomier-Layrargues

**Affiliations:** ^1^Department of Surgery, Centre Hospitalier de l'Université de Montréal, 264, East René-Lévesque Blvd, 3rd floor, Montreal, QC, Canada H2X 1P1; ^2^Department of Radiology, Centre Hospitalier de l'Université de Montréal, 264, East René-Lévesque Blvd, 3rd floor, Montreal, QC, Canada H2X 1P1; ^3^Liver Unit, Centre Hospitalier de l'Université de Montréal, 264, East René-Lévesque Blvd, 3rd floor, Montreal, QC, Canada H2X 1P1

## Abstract

*Background*. Transcatheter arterial lipiodol chemoembolization (TACE) can be used in cirrhotic patients with hepatocellular carcinoma to avoid tumor progression before transplantation. *Objective*. To evaluate the efficacy and safety of TACE used as a bridge to liver transplantation. *Methods*. TACE was performed in 30 cirrhotic patients with hepatocellular carcinoma. Milan criteria were used to select patients for transplant. Patients had a good or moderately impaired liver function, no arterioportal fistulae, and a good portal perfusion. *Results*. 48 TACE were performed in 30 patients. Before transplantation, 4 patients were dropped off the list due to tumor extension or liver failure. Complete necrosis of the tumor was observed in 11 patients and partial necrosis in 15 patients. After transplantation, 6 patients died and tumor recurrence was observed in 5 patients with a tumor beyond Milan criteria or no response to TACE. *Conclusion*. TACE is useful as a bridge to liver transplantation in a selected group of cirrhotic patients with hepatocellular carcinoma. A combined therapeutic approach before surgery might improve the prognosis in these patients.

## 1. Introduction

Incidence of hepatocellular carcinoma (HCC) has increased significantly over the last decades. Worldwide it represents the fifth most common cancer and the third cause of cancer mortality among the general population [1, 2]. Hepatitis B and C and alcohol abuse are major risk factors for HCC. Several treatment modalities exist based on tumor location and size. Among others, liver transplantation was confirmed as a valuable treatment for HCC during the 1990s as it was observed that small incidental HCC discovered in the explanted liver had no adverse impact on posttransplantation outcome [3, 4]. Thus, over the years, orthotopic liver transplantation (OLT) has become the mainstay of treatment for small centrally located HCC in a cirrhotic liver. Since the late nineties, most transplantation centers have agreed to perform liver transplantation for HCC based on Milan criteria. The Milan criteria were elaborated by Figueras et al. who showed that patients with one hepatic lesion smaller than 5 cm or with three lesions smaller than 3 cm can expect a 5-year survival rate after liver transplantation of 70% with recurrence rate below 15% [5]. Those results were supported by several other research groups [6–8]. 

However, the time waiting for an organ represents a major pitfall to liver transplantation. This may lead to tumor progression and subsequent ineligibility for transplantation. In Europe and USA, there are reports that liver transplant waiting lists are getting longer so that a growing number of patients must be removed from the lists due to tumor progression exceeding the accepted transplant criteria [2]. Dropout rates of more than 20% have recently been reported [9].

Transcatheter arterial chemoembolization (TACE) has been proposed as a treatment to bridge the waiting time between the diagnosis and liver transplantation. In some studies, it has been demonstrated that TACE may limit tumor growth, diminish dropout rate, and cause significant tumor necrosis, which may reduce tumor dissemination during surgery and possibly recurrences [10]. However, some studies have also showed opposite results [5, 11, 12]. 

The aim of this study was to evaluate retrospectively the usefulness and tolerance of TACE before OLT in cirrhotic patients with hepatocellular carcinoma.

## 2. Methods

From January 1st 1996 to January 1st 2008, all cirrhotic patients with HCC listed for OLT and treated with TACE at our transplantation center were included for analysis. 

Diagnosis of HCC was made in accordance with the European Association for the Study of the Liver (EASL) guidelines: typical lesions on 2 imaging studies (computed tomographic (CT) scan, magnetic resonance imaging (MRI), ultrasound) or on 1 imaging modality with an elevated serum alfa-fetoprotein over 400 ng/ml or liver biopsy with histologic confirmation of HCC [13].

The selection of patients with HCC for TACE was based on the following inclusion criteria: good or moderately impaired hepatic function (Child Pugh classes A and B), hepatopetal portal blood flow, absence of portal vein thrombosis, absence of arterioportal fistula, absence of extensive portosystemic shunting, and a waiting time for liver transplantation estimated to be more than 3 months. TACE aimed both at limiting tumor progression and downstaging. Downstaging was conducted in a limited number of patients exceeding the Milan criteria. In those patients, TACE treatments were administered until Milan criteria were reached in order to become eligible for OLT.

All the patients signed an informed consent form and the procedures were done in accordance with the Helsinki declaration of 1975.

Selective TACE were performed by interventional angio-radiologists (PP and LB) using cisplatin and lipiodol. Gel Foam (*Pfizer*, Canada) was added to stasis following the injection of the cisplatin-lipiodol solution into the hepatic artery. Tumor response was assessed by CT scan and/or MRI every 3 months post chemoembolization. Changes in the tumor size of at least 5 mm in diameter were considered to be significant. TACE was performed every 3 months according to response or until transplantation if patients were eligible. Child Pugh scores and complications were monitored following every treatment.

All patients transplanted received a cadaveric liver. Explanted livers underwent extensive histologic analysis and staging of HCC. The modified Edmondson scoring system was used to grade the level of differentiation of HCC nodules [14]. 

All patients were followed until death or January 2009. In the follow-up period, surgical complications, hepatitis recurrence, HCC recurrence, and patients survival were analysed.

Categorical data were compared using the Fischer exact test. Hazard ratio associated with the Milan criteria and cancer recurrence were calculated using 95% confidence intervals. Survival rate was calculated for patients with and without cancer recurrence after OLT according to the Kaplan-Meier method, groups were compared using the log rank test. One sided *P*-values <.05 were considered significant. Analysis were done using SAS statistical software (version 8.2).

## 3. Results

### 3.1. Patients Characteristics (Table 1)

A total of 30 patients were included for analysis. There were 23 men and 7 women (mean age: 55 years, range 35–68 years) (22 Caucasians, 4 Asians, 3 South Americans, and 1 African).

Liver cirrhosis was mostly caused by hepatitis C virus (HCV) and/or hepatitis B virus (HBV). Other etiologies of cirrhosis were also identified such as alcohol abuse, nonalcoholic steatohepatitis (NASH), and combined causes (4 HCV  +  alcoholism, 2 HCV + hemochromatosis, 1 HCV + HBV, 1 HBV + alcoholism, 1 hemochromatosis + alcoholism). 

Diagnosis of HCC was based on the presence of typical lesions on 2 imaging studies (18 patients) or on 1 imaging study with elevation of serum alpha-fetoprotein (9 patients), and liver biopsy was done to confirm the diagnosis in 3 patients. 

At the time of diagnosis, the majority of patients had either 1 (60%) or 2 (10%) suspected tumoral nodules (size: 23,8 mm ± 13.7 mm (mean ± SD). A total of 6 patients (20%) exceeded Milan criteria (Table 2).

### 3.2. Listing

All patients analysed were listed for OLT. Thirteen patients (43%) were listed before having TACE and 17 (57%) after. At the time of listing, 24 patients (80%) were within Milan criteria and 6 patients (20%) were not within those criteria. Therefore, in these 6 patients, TACE was a downstaging attempt.

### 3.3. TACE

Forty-eight TACE treatments were administered to the 30 patients (mean 1.6/patient; range 1–7). The majority of interventions were done in Pugh class A patients (40 interventions or 83%) before TACE. Increase in the Pugh class from A to B was observed following 3 TACE (6%).

Complications associated with TACE were divided into minor and major categories (Table 3). Among the 48 TACE procedures, 20 (42%) did not suffer from any complications. A total of 27 interventions (56%) resulted in complications, which were essentially of the minor category (Table 3) mostly abdominal pain and nausea. As for major complications, we identified 2 ischemic cholecystitis, 1 acute liver failure, 1 thrombosis of the femoral artery, 1 splenic infarction, and 1 ischemic atrophy of the left hepatic lobe. In addition, 1 patient died from acute liver failure, sepsis, and acute renal failure thought to be directly related to the TACE procedure.

### 3.4. Radiologic Follow-Up (Table 4)

Radiologic follow-up after TACE treatment showed a decrease of tumoral size of at least 5 mm in 13 patients (43%) (range: 5–34 mm). In 6 patients (20%), tumoral size remained stable (±5 mm) and 5 patients (17%) had an increase of tumoral size of at least 5 mm. New liver lesions were observed in 9 patients (30%) after TACE. Therefore, a total of 10 patients (33%) did not respond to TACE due to tumor progression and/or appearance of new hepatic lesions. There were 6 patients (20%) within Milan criteria at listing that were transplanted before having radiologic follow-up after TACE and thus, radiological response to TACE could not be assessed in these patients.

### 3.5. Downstaging

As mentioned before, downstaging was attempted in 6 patients that were exceeding Milan criteria at HCC diagnosis. Analysis of their radiological response to TACE showed that downstaging could be obtained in 3 of the 6 patients (50%).

### 3.6. Drop-Off (DO)

During the time waiting for OLT, 4 out of 30 patients (13%) were removed from the transplant list. Three patients were excluded because of HCC progression over transplant criteria and 1 died following his first TACE treatment. The mean delay between listing and DO was 109 days (range: 17–171 days). All 4 dropouts received 1 TACE each before exclusion.

### 3.7. Orthotopic Liver Transplantation (OLT) (Table 5)

Twenty-six patients (87%) were transplanted with a mean waiting time on the transplant list of 110 days (range: 42–460 days). At transplantation, 22 patients (85%) met Milan criteria but 4 patients (15%) exceeded Milan criteria; tumor growth from within to beyond Milan criteria after being listed was observed in 2 patients, downstaging failed in 2 others. These patients were transplanted despite being beyond Milan criteria either because the tumor was at the margin of the criteria or because of uncertainty about the malignant nature of some nodules.

### 3.8. Pathology of Explanted Livers

The histopathologic analysis of the 26 explanted livers showed complete tumoral necrosis in 10 cases (42%) and partial necrosis in 15 cases (48%). The mean Edmondson score of patients with viable cancer was 2,5/4. Microvascular invasion was observed in 3 cases and macrovascular invasion in one patient. In the patients transplanted exceeding Milan criteria just before OLT (4 cases), 1 had complete tumoral necrosis (a successful downstaging attempt) and 3 patients (75%) had HCC that remained outside Milan criteria at pathological analysis. 

In the 10 livers where complete HCC necrosis was observed, 9 were within Milan before transplantation. Seven patients had a regression of the tumor following TACE, 2 had stable lesions, and 1 had new lesions identified.

### 3.9. Follow-Up

Table 6 summarizes patients' characteristics after OLT with a mean follow-up of 56 months (range: 6–142 months). At follow-up, we identified 21 patients (81%) without cancer recurrence and 5 with cancer recurrence (19%). In patients identified with cancer recurrence, 3 (60%) had cancer in the transplanted liver and 2 (40%) had lung metastasis. Mean time to recurrence was 13.8 months (range 6–22 months). 

Patients without cancer recurrence had more tumoral regression/stability after TACE than patients with cancer recurrence (71% versus 0%; *P* = .051). In addition, patients with no tumoral recurrence were almost all within Milan criteria before transplantation (90%) as opposed to patients with tumoral recurrence (60%). Therefore, Milan status was associated to a relative risk of cancer recurrence posttransplantation of 6.33 with a 95% CI (1.53–26.18). Patients with no cancer recurrence showed more complete necrosis (48% versus 0%; *P* = .066) and significantly less microscopic invasion (0% versus 60%; *P* = .004) on pathological analysis. 

**Figure 1:**
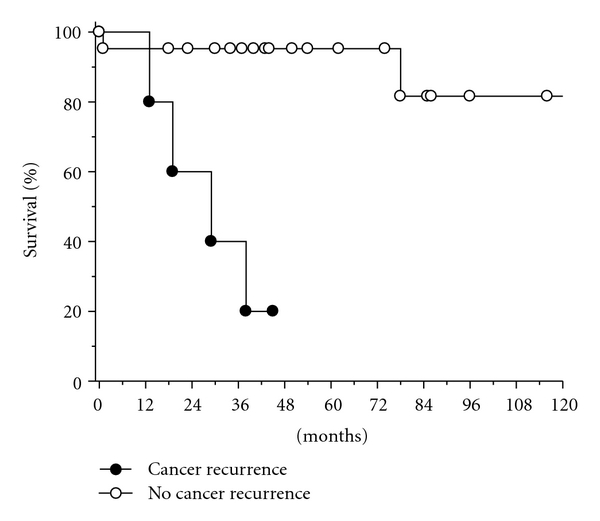
Cumulative proportion of patients surviving after OLT (Kaplan-Meier plot) with and without cancer recurrence. The difference between the 2 groups was significant (*P* < .01; log rank test).

Six patients died (23%) at follow-up: 1 from postoperative complications, 4 from cancer recurrence, and 1 from cirrhosis associated with hepatitis C 7 years posttransplantation (Figure 1). Therefore, survival of patients with tumor recurrence was poor, mortality was significantly lower in patients without cancer recurrence compared to patients with cancer recurrence (10% versus 80%; *P* = .002).

### 3.10. Subgroup Analysis according to Milan Status

In the 4 patients that exceeded Milan criteria before transplantation, 2 represented a failed attempt at downstaging and cancer progression was observed in 2. Three of the 4 patients (75%) exceeding Milan at OLT had HCC discovered at histology. At follow-up, HCC recurrence was observed in 2 of these patients (50%). Hence, being outside Milan before OLT significantly increased risk of cancer recurrence after OLT (RR: 6.33, 95% CI: 1.53–26.18). 

In the 22 patients within Milan criteria before transplantation, 10 (45%) had complete HCC necrosis and all remaining HCC were within Milan criteria at pathology. So far, 3 patients (14%) had HCC recurrence after OLT. Five died (23%), 1 from postoperative complications, 3 from HCC recurrence, and 1 from cirrhosis 7 years after OLT.

## 4. Discussion

Hepatocellular carcinoma represents a serious disease with a high mortality rate. When a tumor is centrally located within an underlying cirrhosis, the best treatment is liver transplantation. However, tumoral size is a major determinant of eligibility for transplantation. Moreover, time waiting for an organ is associated with tumoral growth, which is responsible for most dropouts. Over the years, several approaches including TACE were used to limit tumoral growth in cirrhotic patients on the waiting list and hopefully tumor recurrence after transplantation. Some researchers stated that the response to TACE could be used to predict risks of tumor recurrence after liver transplantation [15–18]. However, these studies are difficult to analyse as they are either using different chemotherapeutic agents or do not include a pathological analysis of explanted livers.

Milan criteria are used in most transplant centers to select patients eligible for liver transplantation. Extended criteria are uncommonly used in some centers, with the most recognized being the San Francisco criteria. It has also been suggested that TACE can be used to downstage the tumor within Milan criteria or within San Francisco criteria before transplantation [19–24].

The present study reports our experience with cirrhotic patients with HCC who underwent TACE and who were listed for OLT between January 1996 and January 2008. Only patients within Milan criteria were eligible for transplantation. We analysed retrospectively tumoral response following TACE treatments, incidence of dropouts on the waiting list, histopathological characteristics of HCC on explanted livers, and evolution of patients after transplantation. Although a small number of patients were analyzed, interesting findings were observed. 

We confirm that TACE can influence tumoral growth in most patients while waiting for transplantation. Forty-eight TACE in 30 patients were performed: a decrease of tumoral size was observed in 13 patients and an increase in 5 patients. Twelve patients had stable lesions. Therefore, among 30 transplant candidates, 26 could be operated, with 4 being dropped out (3 due to tumor progression). Histopathological analysis of the 26 explanted liver showed complete necrosis of HCC in 10, reinforcing the effectiveness of TACE treatments. Subgroup analysis of these patients with no residual carcinoma showed that 9 patients (90%) were within Milan before OLT and that none had cancer progression observed after TACE treatments. 

Despite our intent to transplant only patients within the Milan criteria, 4 patients exceeding these criteria were operated. This emphasizes the difficulty to evaluate correctly the tumoral stage using radiologic imaging. In these four patients, the disease was thought to be at the margin of Milan criteria or the exact nature of some nodules was falsely presumed to be benign. This difficulty of correctly evaluating tumoral size and extension is observed in most transplant centers. 

As previously stated, TACE could be used either to limit tumoral growth within Milan criteria or to downstage HCC. In 6 patients, downstaging was attempted using TACE before transplantation and this was successful for 3 patients (50%). In these patients, the pathological analysis either demonstrated variable level of HCC necrosis (2 patients) or complete necrosis of HCC (1 patient). Follow-up after transplantation showed that none of these 3 patients had tumor recurrence or died. Such effective downstaging within the transplant criteria using TACE without increasing the risk of cancer recurrence has also been reported by other research teams [19–24].

Our results support the use of Milan criteria to select patients for liver transplantation. Patients within Milan treated with OLT had high levels of necrosis on histopathological analysis (45% did not have residual HCC), very low rate of HCC recurrence (14%), and low mortality rate during follow-up (23%), which is in accordance with the literature [19, 20, 23, 24]. Patients exceeding Milan before transplantation had little necrosis on histopathological analysis (75% had viable HCC), high rate of cancer recurrence, and death within 2 years of surgery.

As shown by others [15–18], the response to TACE provides important information for the prognosis after transplantation. A significant number of patients who did not respond to TACE, even if the tumor was still within the Milan criteria, demonstrated postoperative tumoral recurrence. This is best explained by the aggressiveness of the tumor. Therefore, response to TACE could be used as an additional tool in order to better select patients for transplantation [25].

The tolerance to TACE was rather good as previously reported [13] but several major complications occurred after TACE including acute transient hepatic insufficiency, 1 ischemic cholecystitis, femoral artery thrombosis, and splenic infarction; only one patient died from sepsis and acute hepato-renal failure.

These encouraging results must be tempered by the fact that our population was small and highly selected and also by the possible adverse events following TACE. TACE was only performed in patients with good liver function, patent portal vein, and absence of significant portosystemic shunting. Therefore, our conclusions cannot be extrapolated to a larger population.

## 5. Conclusion

In conclusion, the present study confirms the usefulness of TACE as a bridge to liver transplantation in cirrhotic patients with hepatocellular carcinoma within the Milan criteria and also to downstage some highly selected patients. This approach is associated with a good survival rate and a low rate of cancer recurrence. TACE was also well tolerated. These encouraging results could be improved in the future by a better selection of transplant candidates based on more accurate imaging techniques and better assessment of biological characteristics of HCC.

## Figures and Tables

**Table 1:** Baseline patients characteristics.

Variable	*n*
Number of patients	30
Sex (M/F)	23/7
Median age at listing years (range)	55 (35–68)
Origin (%)	
Caucasian	22 (73)
Asian	4 (13)
South American	3 (10)
African	1 (3)
Cause of liver cirrhosis (%)	
Hepatitis C virus	9 (33)
Hepatitis B virus	8 (27)
Combined causes	9 (30)
Alcohol abuse	2 (7)
NASH	2 (7)
Hepatocellular carcinoma (%)	30 (100)
Diagnosis (%)	
2 imaging studies	18 (60)
1 imaging study + alfa-foeto proteins	9 (30)
Biopsy	3 (10)
Median AFP, ng/ml (range)	27,8 (2.6–3000)
Mean lesion size, mm (range)	23,8 (10–60 mm)
Number of lesions, patients (%)	
One lesion	18 (60)
Two lesions	3 (10)
Three lesions	7 (23)
Four lesions	2 (7)
Milan at diagnosis (%)	
In	24 (80)
Out	6 (20)

**Table 2:** Characteristics of hepatocarcinoma outside Milan criteria at diagnosis.

Patient number	Number of lesions	Size of lesions (mm)
# 13	3	45/30/26
# 16	2	50/22
# 18	1	60
# 24	4	45/25/10/10
# 27	1	55
# 31	3	35/22/10

**Table 3:** Adverse events following transarterial chemoembolization (TACE) (48 treatments in 30 patients).

Variable	*n*
Number of TACE/patient (mean, range)	1.6 (1–7)
Adverse events (%)	
None	20 (42)
Yes	28 (58)
Minor events, number of TACE (%)	24 (50)
Pain in right upper quadrant	18 (38)
Nausea/emesis	7 (15)
Chemical arteritis	5 (10)
Corticosteroids-related diabetes unbalances	2 (4)
Ischemic hepatitis	2 (4)
Leucocytosis	1 (2)
Fever	1 (2)
Allergic reaction	1(2)
Major events, number of TACE (%)	7 (15)
Ischemic cholecystitis	2 (4)
Transient acute hepatic failure	2 (4)
Thrombosis of superficial femoral artery	1 (2)
Splenic infarction	1 (2)
Ischemic atrophy of the left hepatic lobe	1 (2)
Acute renal failure	1 (2)*
Spontaneous bacterial peritonitis	1 (2)*
Sepsis	1 (2)*
Death	1 (2)*

*Those events occurred in the same patient.

**Table 4:** Radiologic evolution after transarterial chemoembolization (TACE).

Variable	*n*
Tumor size (%)	
Reduction >5 mm	13 (43)
Stability +/−5 mm	6 (20)
Progression >5 mm	5 (17)
Unknown*	6 (20)
New lesions (%)	
No	15 (50)
Yes	9 (30)
Unknown*	6 (20)
Overall response (%)	
Regression (size reduction >5 mm and no new lesions)	10 (33)
Stability (size stability +/−5 mm and no new lesions)	4 (13)
Progression (size progression and/or new lesions)	10 (33)
Unknown	6 (20)
Downstaging (6 patients)	
Yes	3 (50)
No	3 (50)

*These patients did not have imaging after TACE before transplantation.

**Table 5:** Orthotopic liver transplantation.

Variable	*n*
Patients with OLT/Patients listed for OLT	26/30
Mean waiting time in days (range)	110 (4–460)
Milan at transplantation (%)	
In	22 (85)
Out	4
Progression beyond Milan criteria	2
Failed downstaging within Milan criteria	2

**Table 6:** Characteristics of patients with and without cancer recurrence after OLT (mean follow-up 56 months; range: 6–142 months).

	No cancer recurrence	Cancer recurrence	P*
*n*	21	5	
Tumor response to TACE			
regression/stability (%)	15 (71)	0	.051
Progression	2 (10)	4 (80)	
Not evaluated	4 (19)	1 (20)	
Milan criteria before OLT (%)			
within Milan	19 (90)	3 (60)	.1548
outside Milan	2 (10)	2 (40)	
Pathological tumor analysis (%)			
complete necrosis	10 (48)	0	.066
microscopic invasion	0	3 (60)	.004
Mortality	2 (10)	4 (80)	.002

*One sided *P*-values calculated using the Fisher exact test.
